# Identification of a Risk Stratification Model to Predict Overall Survival and Surgical Benefit in Clear Cell Renal Cell Carcinoma With Distant Metastasis

**DOI:** 10.3389/fonc.2021.630842

**Published:** 2021-03-11

**Authors:** Jiasheng Chen, Nailong Cao, Shouchun Li, Ying Wang

**Affiliations:** ^1^Department of Urology, The Affiliated Changzhou No.2 People's Hospital of Nanjing Medical University, Changzhou, China; ^2^Department of Urology, Shanghai Jiao Tong University Affiliated Sixth People's Hospital, Shanghai Eastern Institute of Urologic Reconstruction, Shanghai Jiao Tong University, Shanghai, China

**Keywords:** clear cell renal cell carcinoma, distant metastasis, nomogram, overall survival, surgical benefit

## Abstract

**Background:** Clear cell renal cell carcinoma (ccRCC) is the main subtype of renal cell carcinoma and has different prognoses, especially in patients with metastasis. Here, we aimed to establish a novel model to predict overall survival (OS) and surgical benefit of ccRCC patients with distant metastasis.

**Methods:** Using data from the Surveillance, Epidemiology, and End Results (SEER) databases, we identified 2185 ccRCC patients with distant metastasis diagnosed from 2010 to 2015. Univariate and multivariate Cox analysis were used to identify significant prognostic clinicopathological variables. By integrating these variables, a prognostic nomogram was constructed and evaluated using C-indexes and calibration curves. The discriminative ability of the nomogram was measured by analyses of receiver operating characteristic (ROC) curve. A risk stratification model was built according to each patient's total scores. Kaplan-Meier curves were performed in the low-, intermediate- and high-risk groups to evaluate the survival benefit of surgery.

**Results:** Eight clinicopathological variables were included as independent prognostic factors in the nomogram: grade, marital status, T stage, N stage, bone metastasis, brain metastasis, liver metastasis, and lung metastasis. The nomogram had a better discriminative ability for predicting OS than Tumor-Node-Metastasis (TNM) stage. The C-index was 0.71 (95% CI 0.68–0.74) in the training cohort. The calibration plots demonstrated that the nomogram-based predictive outcomes had good consistency with the actual prognosis results. Total nephrectomy improved prognosis in both the low-risk and intermediate-risk groups, but partial nephrectomy could only benefit the low-risk group.

**Conclusions:** We constructed a predictive nomogram and risk stratification model to evaluate prognosis in ccRCC patients with distant metastasis, which was valuable for prognostic stratification and making therapeutic decisions.

## Introduction

Renal cell carcinoma (RCC) is one of the most common malignant tumors in the genitourinary system. The latest cancer statistics report illustrated that more than 65,000 patients were diagnosed with RCC in the US, causing more than 15,000 deaths every year (1). Clear cell renal cell carcinoma (ccRCC) is the predominant histology of RCC, representing 75% of all cases (2). Among them, many patients with this disease are diagnosed with locally advanced disease or distant metastases despite improvements in the cancer control and survival rates. Clinically, approximately 16% of ccRCC patients have metastasis at diagnosis, and even one-third of localized ccRCC patients will develop metastatic lesions after tumor resection. The 5-year overall survival (OS) rate of metastatic ccRCC is only 12% (3). For RCC patients with distant metastasis, although the Memorial Sloan-Kettering Cancer Center (MSKCC) criteria and the International Metastatic RCC Database Consortium (IMDC) criteria can be used to evaluate the outcome of patient treatment, the impact of metastatic site and the overall tumor burden on survival is still missing (4, 5). Therefore, more practical tools and concise are required to improve the prognostic prediction of ccRCC patients with distant metastasis.

Cancer metastasis is a multistep process involving complex genetic alterations that drive the transformation of primary tumors into highly malignant and metastatic tumors (6, 7). To successfully metastasize, tumor cells must escape from the primary tumor, intravasate into circulatory and lymphatic systems, avoid immune attack, extravasate at distant capillary beds, and invade and proliferate in distant organs (8–10). For ccRCC, intensive studies demonstrated that different genes mediate tumor cell metastasis to different locations. The common metastasis sites of ccRCC include lung (in 50–60% of patients with metastases), bone (in 30–40%), liver (in 30–40%) and brain (in 5%) (11, 12).

The classic anatomical prognostic system is the tumor (T), node (N), and metastasis (M) classification, which is the most commonly used prognosis-predicting system for ccRCC patients (13). However, the TNM staging system lacks accuracy in predicting the prognostic of ccRCC patients, especially for ccRCC patients with distant metastasis (14). In ccRCC patients with distant metastasis, prognosis is further driven by the site of metastasis and the number of metastatic sites (15, 16). In addition, ccRCC patients with distant metastasis can be affected by clinical prognostic factors, including sex, age, marital status, race, and clinicopathological parameters such as grade, tumor size, and surgery treatment. Therefore, in consideration of all of these clinical factors, it is important to build a comprehensive prognostic model to accurately evaluate the prognosis of each patient. This predictive model can help doctors make therapeutic decisions.

Recently, nomogram has been accepted as a reliable tool to quantify risk by incorporating and evaluating important factors to assess prognostic outcome in multiple cancers (17–19). Several nomograms have been established to predict the risk of RCC recurrence and survival (20–22). However, there is no nomogram to estimate the prognostic outcome of ccRCC patients diagnosed with distant metastasis. In this study, we used data from the Surveillance, Epidemiology, and End Results (SEER) databases to establish and validate a nomogram that estimates the survival of ccRCC patients with distant metastasis.

## Materials and Methods

### Data Source and Patient Selection

Patient data came from the Surveillance, Epidemiology, and End Results (SEER) database, which covers approximately 28% of the US population. In our study, patient selection based on the following inclusion and exclusion criteria. Inclusion criteria: (a) diagnosed between 2010 and 2015; (b) molecular subtype of clear cell carcinoma; and (c) diagnosed initially with at least one distant metastatic site. Exclusion criteria: (a) unknown metastatic status; (b) age at diagnosis under18 years; (c) incomplete demographic and clinical data, including race, marital status, T/N stage and grade; and (d) missing follow-up data.

### Nomogram Construction and Validation

We randomly divided the patients diagnosed from 2010 to 2013 into two cohorts, the training cohort and the validation I cohort, with a ratio of three to one, and we assigned the patients diagnosed from 2014 to 2015 as the validation II cohort. Categorical variables in the three cohorts were presented as frequencies and proportions. Univariate Cox regression analyses were used to calculate the influence of each variable on OS. Significant prognostic factors identified from the univariate analysis were further analyzed in a multivariate Cox proportional hazard model, and the corresponding 95% confidence interval (CI) for each potential risk factor was calculated. Based on the result of the multivariate model, a nomogram was built to predict 1-, 2- and 3-year OS. The discriminative ability of the nomogram was measured using the 1-, 2-, and 3-year survival area under curve (AUC) values from time-dependent receiver operating characteristic (ROC) curves. Predictive accuracy was assessed using the concordance index (C-index) and calibration plot. Additionally, a risk stratification model was established on the basis of each patient's total score in the nomogram, and all patients were divided into three prognostic groups.

### Statistical Analyses

Univariate and multivariate Cox regression analyses were performed to identify the prognostic factors. Kaplan-Meier curves was used to estimate the OS. The significance of differences in OS was assessed by log-rank test. Cox regression analysis, Kaplan-Meier curves, and the log-rank test were conducted by the *glmnet* and *survival* packages. The nomogram was established with the *rms* and *survival* packages. All statistical analyses were performed in R studio (version 3.6.2), and statistical significance was set at a *p*-value of <0.05.

## Results

### Demographic and Clinical Characteristics of Patient Patients

Overall, 2,185 ccRCC patients with distant metastasis were included in this study. Among all patients, 1,027, 342, and 816 subjects were assigned to the training, validation I and validation II cohorts, respectively. The demographic and clinical characteristics of patients in each subgroup are demonstrated in Table 1. There was no significant difference in the distribution of the number of patients in different cohorts. Generally, most patients were male (1,523; 69.7%), aged 60–79 years (1,180; 54.0%), married (1,493; 68.3%), and white (1,891; 86.5%). Moreover, most patients underwent total nephrectomy (1,765; 80.8%).

**Table 1:** Demographic and clinical characteristics of ccRCC patients with distant metastasis.

	**Training cohort (*N* = 1,027)**	**Validation I cohort (*N* = 342)**	**Validation II cohort (*N* = 816)**	**Overall (*N* = 2,185)**
**Sex**				
Male	740 (72.1%)	231 (67.5%)	552 (67.6%)	1,523 (69.7%)
Female	287 (27.9%)	111 (32.5%)	264 (32.4%)	662 (30.3%)
**Age (year)**				
18–39	11 (1.1%)	2 (0.6%)	11 (1.3%)	24 (1.1%)
40–59	448 (43.6%)	143 (41.8%)	280 (34.3%)	871 (39.9%)
60–79	521 (50.7%)	180 (52.6%)	479 (58.7%)	1,180 (54.0%)
≥80	47 (4.6%)	17 (5.0%)	46 (5.6%)	110 (5.0%)
**Marital status**				
Married	704 (68.5%)	229 (67.0%)	560 (68.6%)	1,493 (68.3%)
Divorced/Separated	111 (10.8%)	28 (8.2%)	77 (9.4%)	216 (9.9%)
Widowed	65 (6.3%)	38 (11.1%)	62 (7.6%)	165 (7.6%)
Single	147 (14.3%)	47 (13.7%)	117 (14.3%)	311 (14.2%)
**Race**				
White	889 (86.6%)	304 (88.9%)	698 (85.5%)	1,891 (86.5%)
Black	51 (5.0%)	20 (5.8%)	52 (6.4%)	123 (5.6%)
Other	87 (8.5%)	18 (5.3%)	66 (8.1%)	171 (7.8%)
**Grade**				
I	24 (2.3%)	14 (4.1%)	20 (2.5%)	58 (2.7%)
II	262 (25.5%)	92 (26.9%)	181 (22.2%)	535 (24.5%)
III	458 (44.6%)	139 (40.6%)	340 (41.7%)	937 (42.9%)
IV	283 (27.6%)	97 (28.4%)	275 (33.7%)	655 (30.0%)
**T stage**				
T1	131 (12.8%)	60 (17.5%)	116 (14.2%)	307 (14.1%)
T2	169 (16.5%)	69 (20.2%)	137 (16.8%)	375 (17.2%)
T3	649 (63.2%)	185 (54.1%)	493 (60.4%)	1,327 (60.7%)
T4	78 (7.6%)	28 (8.2%)	70 (8.6%)	176 (8.1%)
**N stage**				
N0	793 (77.2%)	259 (75.7%)	619 (75.9%)	1,671 (76.5%)
N1	137 (13.3%)	47 (13.7%)	111 (13.6%)	295 (13.5%)
N2	97 (9.4%)	36 (10.5%)	86 (10.5%)	219 (10.0%)
**Bone metastasis**				
No	719 (70.0%)	236 (69.0%)	570 (69.9%)	1,525 (69.8%)
Yes	308 (30.0%)	106 (31.0%)	246 (30.1%)	660 (30.2%)
**Brain metastasis**				
No	919 (89.5%)	318 (93.0%)	730 (89.5%)	1,967 (90.0%)
Yes	108 (10.5%)	24 (7.0%)	86 (10.5%)	218 (10.0%)
**Liver metastasis**				
No	918 (89.4%)	294 (86.0%)	733 (89.8%)	1,945 (89.0%)
Yes	109 (10.6%)	48 (14.0%)	83 (10.2%)	240 (11.0%)
**Lung metastasis**				
No	409 (39.8%)	139 (40.6%)	287 (35.2%)	835 (38.2%)
Yes	618 (60.2%)	203 (59.4%)	529 (64.8%)	1,350 (61.8%)
**Size (mm)**				
Size ≤ 40	63 (6.1%)	23 (6.7%)	50 (6.1%)	136 (6.2%)
40 < Size ≤ 70	220 (21.4%)	84 (24.6%)	191 (23.4%)	495 (22.7%)
70 < Size ≤ 100	358 (34.9%)	108 (31.6%)	283 (34.7%)	749 (34.3%)
Size > 100	386 (37.6%)	127 (37.1%)	292 (35.8%)	805 (36.8%)
**Surgery**				
No	143 (13.9%)	53 (15.5%)	150 (18.4%)	346 (15.8%)
Partial	31 (3.0%)	12 (3.5%)	31 (3.8%)	74 (3.4%)
Total	853 (83.1%)	277 (81.0%)	635 (77.8%)	1,765 (80.8%)

In total, 30.2% (660), 10.0% (218), 11.0% (240), and 61.8% (1,350) of the patients had bone metastasis, brain metastasis, liver metastasis and lung metastasis, respectively. Additionally, 14.1% (307), 17.2% (375), 60.7% (1,327) and 8.1% (176) of the patients had stage T1, T2, T3, and T4 tumors, respectively. Furthermore, 76.5% (1,671) of the patients were negative for lymphatic metastasis, and 13.5% (295) and 10.0% (219) had N1 and N2 stage.

### Independent Prognostic Factors in the Training Set

Through univariate analysis and subsequent multivariate Cox analysis, marital status (divorced/separated: HR 1.219, 95% CI 0.971–1.531; widowed: HR 1.690, 95% CI 1.277–2.235; single: HR 1.094, 95% CI 0.889–1.347; married as a reference), grade (II: HR 1.126, 95% CI 0.931–1.363; III: HR 1.365, 95% CI 0.809–2.303; IV: HR 1.499, 95% CI 1.217–1.847; I as a reference), T stage (T2: HR 1.354, 95% CI 0.949–1.932; T3: HR 1.388, 95% CI 1.031–1.869; T4: HR 1.626, 95% CI 1.107–2.389; T1 as a reference), N stage (N1: HR 1.934, 95% CI 1.583–2.362; N2: HR 2.375, 95% CI 1.877–3.004; N0 as a reference), bone metastasis (metastasis: HR 1.621, 95% CI 1.378–1.907; no metastasis as a reference), brain metastasis (metastasis: HR 2.158, 95% CI 1.730–2.693; no metastasis as a reference), liver metastasis (metastasis: HR 1.538, 95% CI 1.217–1.943; no metastasis as a reference), and lung metastasis (metastasis: HR 1.709, 95% CI 1.454–2.008; no metastasis as a reference) were found to be statistically significant factors for OS, as shown in Table 2.

**Table 2:** Univariate and multivariate Cox analyses of overall survival in the training set.

	**Univariate analysis**		**Multivariate analysis**	
	**HR (95%CI)**	***P*-value**	**HR (95%CI)**	***P*-value**
**Sex**		0.113		
Female	Reference			
Male	1.135 (0.971, 1.327)			
**Age (years)**		0.118		
18–39	Reference			
40–59	1.214 (0.541, 2.722)	0.638		
60–79	1.254 (0.560, 2.809)	0.582		
≥80	1.753 (0.743, 4.137)	0.200		
**Marital status**		0.036		0.044
Married	Reference		Reference	
Divorced/Separated	1.143 (0.913, 1.430)	0.244	1.219 (0.971, 1.531)	0.088
Widowed	1.443 (1.095, 1.902)	0.009	1.690 (1.277, 2.235)	<0.001
Single	1.145 (0.933, 1.405)	0.194	1.094 (0.889, 1.347)	0.394
**Race**		0.166		
White	Reference			
Black	1.042 (0.754, 1.439)	0.804		
Other	0.811 (0.622, 1.059)	0.124		
**Grade**		<0.001		<0.001
I	Reference		Reference	
II	1.268 (1.057, 1.522)	0.011	1.126 (0.931, 1.363)	0.222
III	1.221 (0.732, 2.037)	0.445	1.365 (0.809, 2.303)	0.244
IV	1.817 (1.493, 2.212)	<0.001	1.499 (1.217, 1.847)	<0.001
**T stage**		<0.001		0.016
T1	Reference		Reference	
T2	1.448 (1.095, 1.916)	0.009	1.354 (0.949, 1.932)	0.094
T3	1.561 (1.232, 1.978)	<0.001	1.388 (1.031, 1.869)	0.031
T4	2.349 (1.616, 3.253)	<0.001	1.626 (1.107, 2.389)	0.013
**N stage**		<0.001		<0.001
N0	Reference		Reference	
N1	2.100 (1.724, 2.558)	<0.001	1.934 (1.583, 2.362)	<0.001
N2	2.454 (1.955, 3.081)	<0.001	2.375 (1.877, 3.004)	<0.001
**Bone metastasis**		0.013		<0.001
No	Reference		Reference	
Yes	1.213 (1.042, 1.412)	0.013	1.621 (1.378, 1.907)	<0.001
**Brain metastasis**		<0.001		<0.001
No	Reference		Reference	
Yes	2.063 (1.664, 2.559)	<0.001	2.158 (1.730, 2.693)	<0.001
**Liver metastasis**		0.010		<0.001
No	Reference		Reference	
Yes	1.343 (1.073, 1.680)	0.010	1.538 (1.217, 1.943)	<0.001
**Lung metastasis**		<0.001		<0.001
No	Reference		Reference	
Yes	1.554 (1.340, 1.803)	<0.001	1.709 (1.454, 2.008)	<0.001
**Tumor size (mm)**		0.023		0.261
Size ≤ 40	Reference		Reference	
40 < Size ≤ 70	1.040 (0.743, 1.454)	0.820	0.993 (0.706, 1.397)	0.969
70 < Size ≤ 100	1.367 (0.995, 1.879)	0.054	1.002 (0.700, 1.432)	0.994
Size > 100	1.274 (0.928, 1.750)	0.135	0.855 (0.600, 1.218)	0.385

### Nomogram Construction and Validation

**Figure 1:**
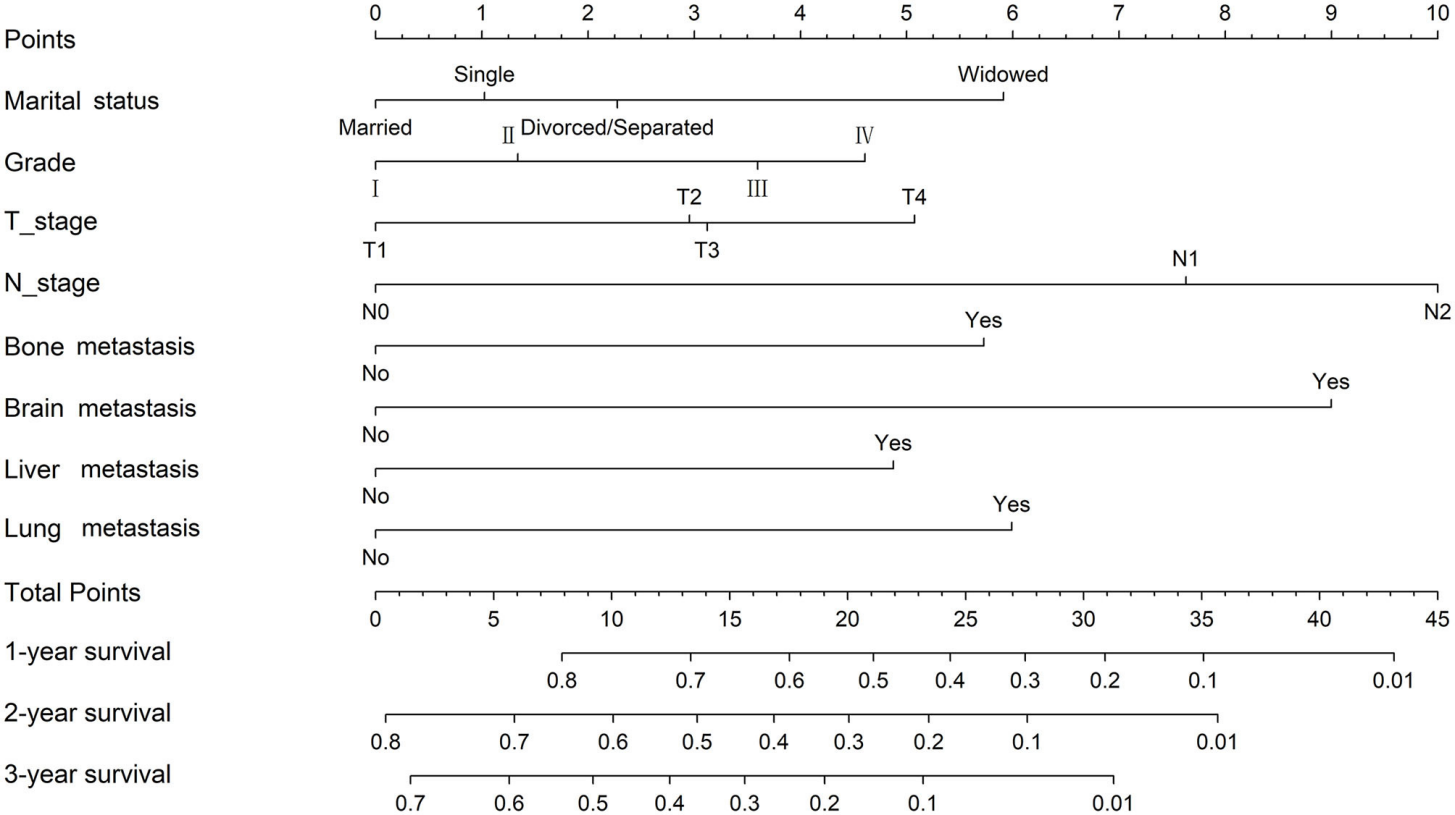
Nomogram for the prediction of 1-, 2- and 3-year overall survival in ccRCC patients with distant metastasis.

Considering the outcomes of the univariate and multivariate Cox regression analyses for OS, eight independent factors in the training cohort were included in the nomogram to predict the 1-, 2-, and 3-year OS rates (Figure 1). Among all included factors, N stage made the most significant contribution to the survival outcome, closely followed by brain metastasis. In addition, marital status, grade, T stage, and the presence of bone/liver/lung metastasis had a moderate impact on prognosis. The 1-, 2- and 3-year survival probabilities of each patient were obtained by adding the score of every prognostic factor.

**Figure 2:**
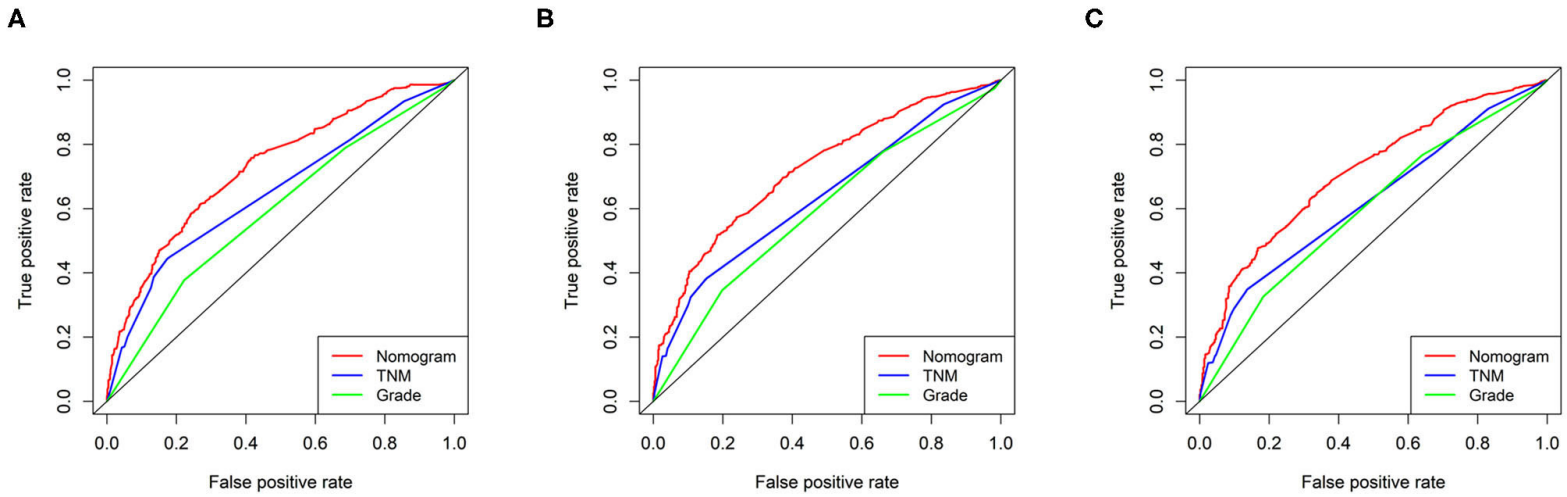
ROC curves of the ability of the nomogram, TNM staging and grade to predict 1-, 2- and 3-year overall survival in the training cohort. **(A)** 1 year time-dependent ROC curve. **(B)** 2 year time-dependent ROC curve. **(C)** 3 year time-dependent ROC curve.

**Figure 3:**
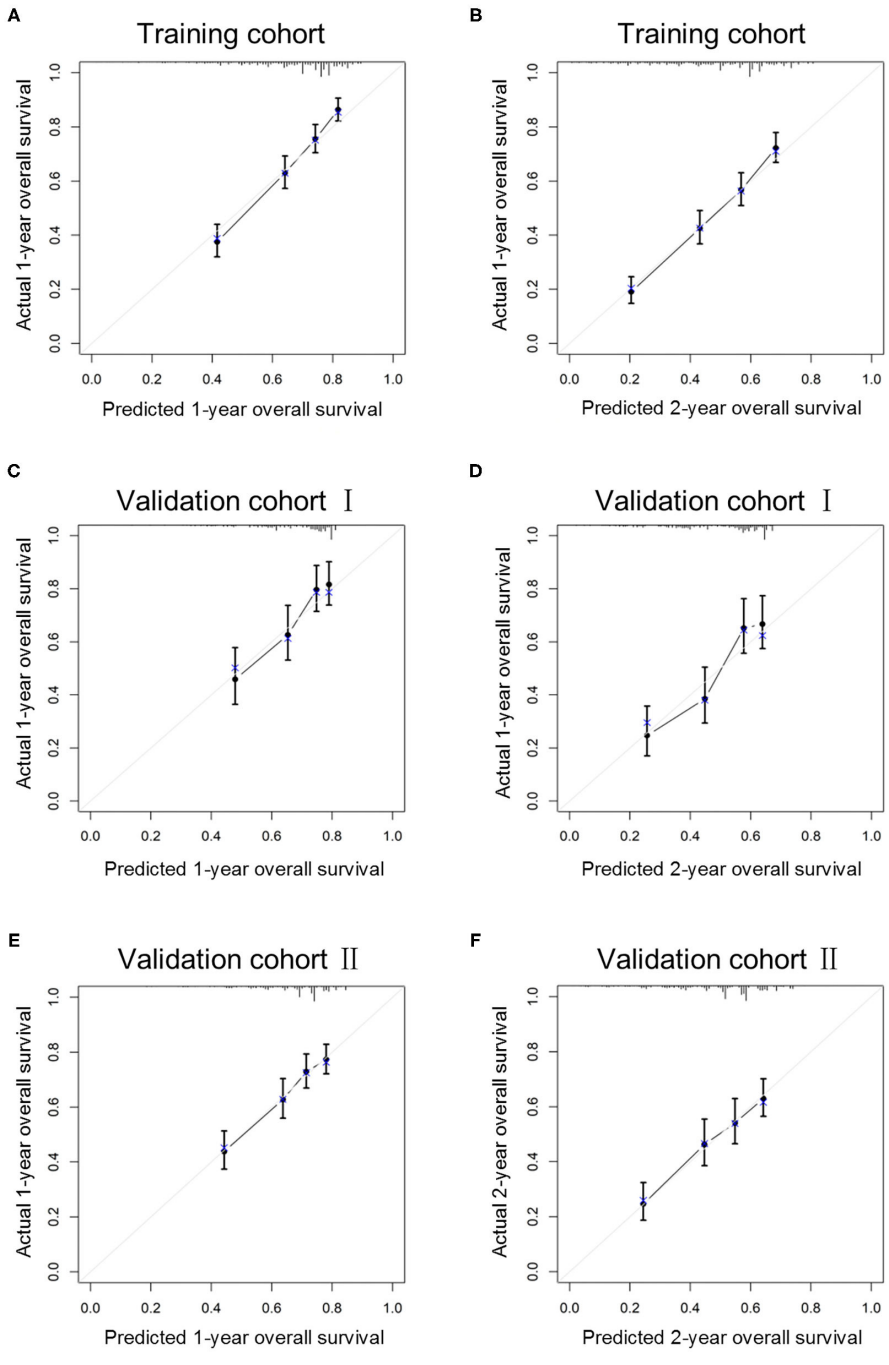
Calibration curves of the ability of the nomogram to predict 1-year **(A)** and 2-year **(B)** overall survival in the training cohort, 1-year **(C)** and 2-year **(D)** overall survival in validation I cohort and 1-year **(E)** and 2-year **(F)** overall survival in validation II cohort.

The C-index in the training cohort (0.71, 95% CI 0.68–0.74) indicated reasonable predictive accuracy of the model. The discriminative ability of the nomogram was measured using the 1-, 2-, and 3-year survival AUC values from time-dependent ROC curve. In the training cohort, the nomogram was significantly superior to TNM staging or grade (1-year AUC: nomogram 0.73 vs. TNM 0.65 or grade 0.59; 2-year AUC: nomogram 0.72 vs. TNM 0.64 or grade 0.59; 3-year AUC: nomogram 0.71 vs. TNM 0.62 or grade 0.60; Figure 2). In addition, in a validation cohort containing both the validation I + II cohorts, the nomogram AUC values for 1-, 2-, and 3-year survival were 0.67, 0.69, and 0.68, respectively. Moreover, the calibration plots in the training and validation cohorts demonstrated that the nomogram-based predictive results were mostly consistent with the actual prognosis results (Figure 3).

### Risk Stratification Model and Survival Benefit of Surgery

**Figure 4:**
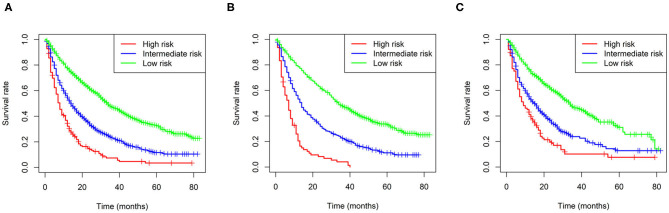
Kaplan-Meier curves of the low-, intermediate- and high-risk groups in all cohorts **(A)**, the training cohort **(B)**, and validation I + II cohort **(C)**.

In addition, we built a risk stratification model based on each patient's total scores in the nomogram. According to the risk stratification model, all the patients were divided into three groups: low-risk group (1,289/2,185, 60.0%; total score < 15), intermediate-risk group (717/2,185, 32.8%; 15 ≤ total score < 25), and high-risk group (1,128/2,185, 51.6%, total score ≥ 25). Kaplan-Meier curves were performed in all cohorts and demonstrated that the risk stratification model can accurately distinguish survival in the three prognostic groups (Figure 4).

**Figure 5:**
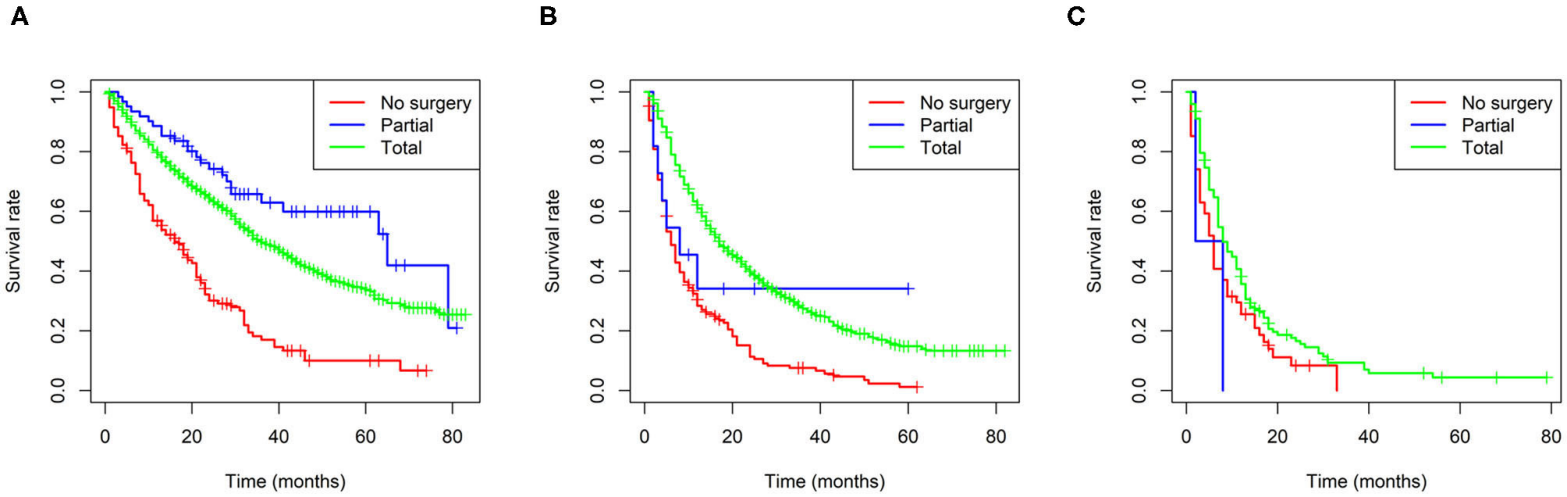
Survival benefit of surgery in the low-risk **(A)**, intermediate-risk **(B)**, and high-risk **(C)** groups.

Furthermore, Kaplan-Meier curves were also performed in the stratified risk groups to assess the survival benefit of surgery (Figure 5). The results indicated that total nephrectomy could prolong overall survival in both the low- and intermediate-risk groups (*p* < 0.0001 and *p* < 0.0001, respectively); however, partial nephrectomy could only benefit the low-risk group (*p* < 0.0001). Interestingly, the low-risk group patients could benefit more in terms of prognosis from partial nephrectomy than total nephrectomy (p = 0.022). However, in the high-risk group, neither total nor partial nephrectomy could significantly improve the prognosis of patients.

## Discussion

In this study, a nomogram was constructed and verified for predicting OS in 2,185 ccRCC patients with distant metastasis from the SEER database. We identified eight demographic and clinical characteristics as prognostic factors, including marital status, grade, T stage, N stage and bone, brain, liver and lung metastasis. In addition, the ROC curves and calibration curves demonstrated favorable discrimination and calibration. Moreover, we built a risk stratification model based on the total score of each patient in the nomogram, and analyzed the survival benefits of surgery choices in the classified risk groups. To our knowledge, this is the first large-cohort, comprehensive retrospective study to construct a nomogram for predicting the prognosis of ccRCC patients with distant metastasis. This predictive tool can be easily applied in clinical practice to predict the survival probability of each patient and help clinicians develop optimal therapy strategies for patients.

Regarding demographic features, marital status was an independent prognostic factor, which is consistent with previous studies (22, 23). Marriage may have a beneficial effect on RCC patients, as it can be associated with support from the spouse, such as helping in activities of daily life and medication reminders. The clinical characteristics grade, T stage, N stage and bone, brain, liver, and lung metastasis were significant for predicting overall survival. Among the sites of metastasis, brain metastasis was the worst factor affecting the prognosis, followed by lung, bone and liver metastasis. Consistently, previous studies have shown that the prognosis of patients with brain metastases is worse than that of patients without brain metastasis (24, 25). However, Abdel-Rahman (26) reported that metastatic RCC patients with liver metastasis seem to have worse outcomes than patients with other sites of metastasis. One explanation is that we mainly focused on clear cell histology rather than all subtypes of RCC. Therefore, the result must be further validated in many ongoing randomized studies.

According to the results of randomized controlled trials, cytoreductive nephrectomy has become the preferred treatment for metastatic RCC patients in the era of cytokine therapy, especially in patients with good performance status (27, 28). In 2005, the molecular-targeted agent sorafenib was approved for the treatment of advanced RCC, opening a new era of molecular-targeted therapy. Clinical data reported so far have clearly demonstrated that, compared with the era of cytokine therapy, the introduction of targeted therapy has significantly improved the prognosis of patients with metastatic RCC (29). However, in the era of targeted therapy, the role of cytoreductive nephrectomy in treating metastatic RCC has been brought into question. The result of CARMENA clinical trial showed that sunitinib alone was not inferior to nephrectomy followed by sunitinib in patients with intermediate- and high-risk metastatic RCC (30). Moreover, from a molecular genetic viewpoint, this intervention can only eliminate the easiest adversary (the main tumor) but cannot prevent cancer-related death. Therefore, the benefits and risks of cytoreductive nephrectomy must be carefully considered. Surgery may not be beneficial if treatment-induced morbidity would substantially affect the patient's quality of life. Thus, demographic and clinical characteristics need to be considered critically to make an optimal decision for each patient. Our study found that total nephrectomy could improve OS in both the low- and intermediate-risk groups, and partial nephrectomy could benefit only the low-risk group, which provides more accurate information for therapeutic decisions.

To our knowledge, this is the first study to generate a predictive nomogram for ccRCC patients with distant metastasis. Although Zheng et al. recently constructed a nomogram for patients with metastatic RCC by combining clinical and pathological characteristics derived from the SEER database (31). In our study, we only included patients with metastatic ccRCC and we stratified the age and tumor size of all patients. In addition, we constructed a training cohort and two validation cohorts to better verify the predictive ability of the nomogram. Moreover, we established a risk stratification model on the basis of each patient's total score from the nomogram and survival benefits of surgery was analyzed in the classified risk groups. As we all know, in the past years both MSKCC and IMDC scores were used almost exclusively to define prognosis of patients with metastatic RCC. Even in the most recent immunotherapy era, their prognostic role was confirmed again and a potential predictive role has emerged (32, 33). Considering that the variables contributing to the IMDC or MSKCC risk model were not registered in the SEER database, there is no comparison in predictive accuracy was conducted between our nomogram and these two models. However, the predictive model proposed in our study is a nomogram, demonstrated to predict the OS more precisely. Regarding to the role of our model in immunotherapy era, it needs to be verified in further study.

The current study has several limitations that should be considered. First, the nomogram was built retrospectively using the SEER database, and it would be better if the nomogram could be verified in a prospective cohort or a clinical trial. Second, the database only contained information on distant metastasis. Some patients may have developed metachronous metastasis during follow-up, and such data are not available from the database. Third, we only focused on patients with ccRCC, and further studies are required to evaluate whether this nomogram is applicable to patients with other histological subtypes. In addition, there is a lack of information about the details of systemic treatment received. This is particularly important given the evidence-based role of targeted therapies in improving the outcomes of metastatic RCC. Finally, patients with missing data with respect to each of the variables were excluded from our cohort, which may lead to potential selection bias. Therefore, further prospective studies are necessary.

## Conclusions

We constructed a novel predictive nomogram and risk stratification model to predict the individual survival of ccRCC patients with distant metastasis. This prognostic model could assist clinicians to identify high-risk patients and make more individualized treatments for patients with different prognoses.

## Data Availability Statement

The original contributions presented in the study are included in the article/supplementary material, further inquiries can be directed to the corresponding author/s.

## Author Contributions

YW and SL designed, conceived this study, and revised the paper. JC contributed to the literature search. JC and NC were involved in data extraction and wrote the manuscript. NC analyzed the data. All authors have approved the final edition of the manuscript.

## Conflict of Interest

The authors declare that the research was conducted in the absence of any commercial or financial relationships that could be construed as a potential conflict of interest.
